# Photomorphogenesis and Photosynthetic Traits Changes in Rice Seedlings Responding to Red and Blue Light

**DOI:** 10.3390/ijms241411333

**Published:** 2023-07-12

**Authors:** Maofei Ren, Shanzhen Liu, Chengzhu Tang, Guiling Mao, Panpan Gai, Xiaoli Guo, Huabin Zheng, Qiyuan Tang

**Affiliations:** 1 College of Agronomy, Hunan Agricultural University, Changsha 410128, China; renmaofei2015@163.com (M.R.); 13461625561@163.com (S.L.); 15226333667@163.com (C.T.); 13665514693@163.com (P.G.); 2 College of Horticulture, Shanxi Agricultural University, Jinzhong 030801, China; mao1925981573@163.com; 3 College of Agronomy, Henan Agricultural University, Zhengzhou 450046, China; 18838933855@163.com

**Keywords:** rice, light quality, red light, blue light, photomorphogenesis, photosynthetic

## Abstract

The purpose of this study is to determine the effects of red and blue lights on the photomorphogenesis and photosynthetic traits of rice seedlings. The rice seedlings were cultured with red light (R), blue light (B), combined red and blue lights (R3B1/R1B1/R1B3), and white light (CK) as the control. The combined application of red and blue lights could promote the growth of rice seedlings to varying degrees; enhance photosynthesis by increasing the seedling leaf area, chlorophyll content, and chlorophyll fluorescence; improve root characteristics by increasing root number, root volume, and root activity; and thus increase the dry matter accumulation of rice seedlings. In addition, the combination of red and blue lights could regulate the expression of genes related to photosynthesis in rice leaves, affect the activity of the Rubisco enzyme, and then affect the photosynthesis of rice seedlings. These results indicate that red and blue lights have direct synergistic effects, which can regulate the growth of rice seedlings and promote the morphogenesis of rice seedlings. The combined application of red and blue lights can be used to supplement the light in rice-factory seedling raising.

## 1. Introduction

In the southern province of China, double-cropping rice is vigorously developed to increase the multiple-cropping index to ensure the yield of rice. In the production of double-cropping rice, people often adopt the method of seedling raising and transplanting to ensure that double-cropping rice has a long enough growth period. With the rapid development of modern agricultural science and technology, plant-factory seedling raising (PFSR) (Figure S1A,B) production has become a key technology in protected rice seedling-raising production. Compared with traditional rice seedling production methods (Figure S1C,D), PFSR is the cultivation of high-quality rice seedlings in an environment that can be controlled at any time and is not affected by external climate change. PFSR can greatly improve the land utilization rate and shorten the cycle of seedling raising [1,2]. In recent years, PFSR has been widely used for fruits, flowers, vegetables, Chinese herbal medicine, rare forest trees, and cash [3,4,5]. However, an inadequate light environment during PFSR often causes poor seedling growth [1]. Therefore, to make the rice seedlings stronger, reasonable light-supplement measures are vitally important for PFSR.

The photo-environment affects the process of crop growth and development through light duration, intensity, and quality [6,7,8,9,10,11]. Light quality is a signal factor that can promote seed germination, plant tissue differentiation, and flower-bud formation [12,13,14,15,16]. Light quality is a kind of regulatory energy that carries different levels of energy through different bands of light and affects the photomorphogenesis, photosynthetic traits, and development of crops [17]. Red light can promote cell proliferation, plant height and leaf elongation, and contribute to dry matter accumulation [11,18,19,20]. Blue light can increase the photosynthetic capacity per unit leaf area, which is beneficial to control plant height, leaf form index, and dry matter accumulation [11,21,22,23]. The combination of red and blue lights can effectively regulate the photomorphogenesis, photosynthetic traits, and development of crops. Adding proper blue light to red light can effectively promote stem thickness, improve photosynthetic efficiency, and cultivate strong crop seedlings [24,25,26]. Although a lot of research exists on the regulation of light quality for crop growth, most of it focuses on horticultural crops, and there is relatively few research on the regulation of light quality for rice growth.

In this study, the effects of red, blue, and combined red and blue lights on the photomorphogenesis and photosynthetic traits of rice seedlings (two rice varieties: XZX24: Xiangzaoxian24 and HZY261: Huazheyou261) are studied. The aim of this study is to evaluate the effects of red, blue, and combined red and blue lights on the photosynthetic characteristics of rice seedlings and to expand the theoretical and technical bases of PFSR production.

## 2. Results

### 2.1. Plant Height, First Leaf Sheath Length, and Stem Width

**Figure 1:**
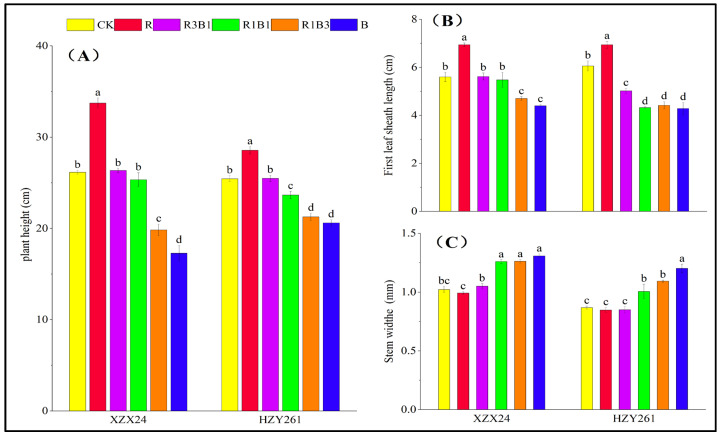
Effects of red, blue, and combined red and blue lights on plant height, first leaf sheath length, and stem width of rice seedlings. (**A**): Plant height, (**B**): first leaf sheath length, (**C**): stem width, CK: white light, R: red light, R3B1: 75% red light + 25% blue light, R1B1: 50% red light + 50% blue light, R1B3: 25% red light + 75% blue light, B: blue light. According to the LSD test, different lowercase letters indicate statistical differences at the 5% level between different light-quality treatments of the same variety.

There were significant differences in plant height, first leaf sheath length, and stem width among different varieties and light-quality treatments (Figure 1). Red light promoted the stem elongation of rice seedlings, and the plant height and first leaf sheath length of XZX24 (HZY261) rice seedlings under red-light treatment significantly increased by 28.89% and 23.92% (12.26% and 14.52%) (Figure 1A,B) compared with white-light treatment, respectively. Blue light promoted an increase in the stem width of rice seedlings, and the stem width of XZX24 (HZY261) rice seedlings under blue-light treatment significantly increased by 27.98% (38.48%) (Figure 1C) compared with white-light treatment.

### 2.2. Leaf Growth

There were significant differences in leaf growth among different rice varieties and light-quality treatments (Table 1). Red light promoted the leaf elongation of rice seedlings, and the leaf length of XZX24 (HZY261) rice seedlings under red-light treatment significantly increased by 18.04% (18.83%) compared with white-light treatment. However, the effects of red, blue, and combined red and blue lights on the leaf width of rice seedlings of XZX24 (HZY261) showed the opposite trend to that of leaf length, with the maximum values for B treatment and the minimum values for R treatment. Nevertheless, the effect of different light qualities on the length–width ratio of rice seedlings of XZX24 and HZY261 showed the same trend to that of leaf length, with the maximum values for R treatment and the minimum values for B treatment. The leaf area of XZX24 presented the maximum values for R3B1 treatment and was significantly larger than the other treatments. Compared with CK, the leaf area of XZX24 treated with R3B1 was significantly increased by 8.57%. Nevertheless, the leaf area of HZY261 had the maximum values for R treatment and was larger than the other treatments. Compared with CK, an increase in the leaf area of HZY261 during R treatment was increased by 11.90%.

### 2.3. Root Growth

There were significant differences in root growth among different rice varieties and light-quality treatments (Table 2), and the root growth parameters of XZX24 were better than HZY261. Among all light-quality treatments, the root growth parameters (root length, root surface area, root volume, and root diameter) of combined red and blue lights treatment were larger than those of white-light, red light and blue light treatments. In addition, the root growth parameters of XZX24 were the largest under R3B1 treatment, while those of HZY261 were the largest under R1B1 treatment. XZX24 (HZY261) showed larger root growth parameters under red light rather than blue light.

### 2.4. Root Activity

**Figure 2:**
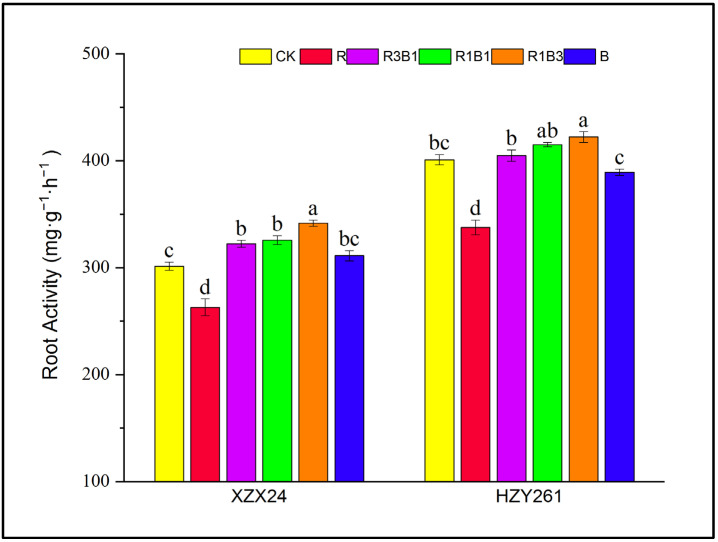
Effects of red, blue, and combined red and blue lights on root activity of rice seedlings. CK: white light, R: red light, R3B1: 75% red light + 25% blue light, R1B1: 50% red light + 50% blue light, R1B3: 25% red light + 75% blue light, B: blue light. According to the LSD test, different lowercase letters indicate statistical differences at the 5% level between different light-quality treatments for the same variety.

There were significant differences in the root activity among different rice varieties and light-quality treatments (Figure 2), and the root activity of HZY261 was better than XZX24. Blue light promoted the root activity of rice seedlings, and the root activity of XZX24 (HZY261) rice seedlings under blue-light treatment significantly increased by 18.37% (15.31%) compared with the red-light treatment. The root activity of XZX24 (HZY261) reached the maximum value under the treatment of R1B3.

### 2.5. Fresh Weight, Dry Weight

**Figure 3:**
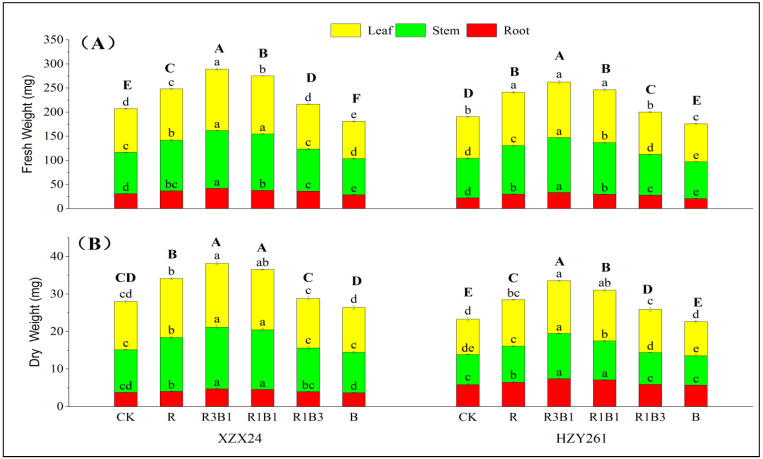
Effects of red, blue, and combined red and blue lights on fresh and dry weight of rice seedlings. (**A**): Fresh weight, (**B**): dry weight, CK: white light, R: red light, R3B1: 75% red light + 25% blue light, R1B1: 50% red light + 50% blue light, R1B3: 25% red light + 75% blue light, B: blue light. Different lowercase letters (capital letters) denote statistical differences among treatments of a cultivar root weight, stem weight and leaf weight (total weight) at the 5% level according to LSD test. Error bars above mean indicate standard error.

There were significant differences in the fresh and dry weights among different rice varieties and light-quality treatments (Figure 3), and the fresh and dry weights of XZX24 were better than HZY261. Among all the light-quality treatments, the fresh and dry weights of the combined red and blue light treatment were heavier than those of white-light, red light and blue light treatments. In addition, the fresh and dry weights of XZX24 (HZY261) were the heavier under R3B1 treatment, and the fresh and dry weights of XZX24 (HZY261) rice seedlings under R3B1 treatment significantly increased by 39.46% and 36.39% (37.95% and 44.20%) compared with white-light treatment, respectively. XZX24 (HZY261) showed the heaviest fresh and dry weights under red-light rather than blue-light treatment.

### 2.6. Pigment Content, SPAD

**Figure 4:**
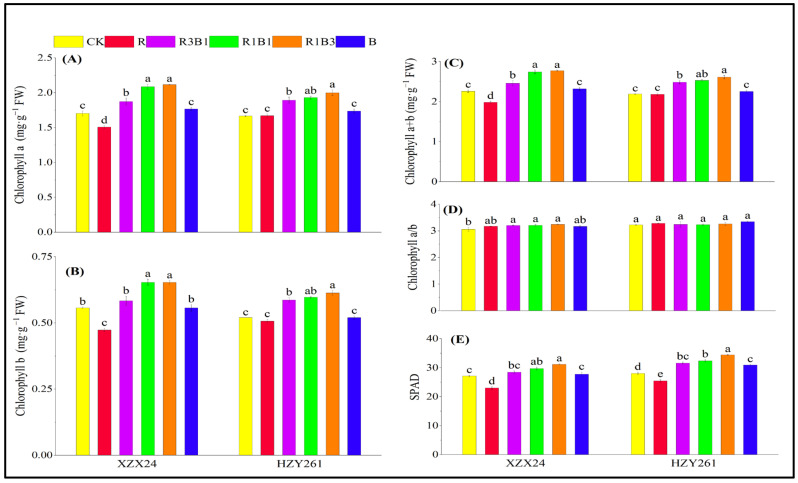
Effects of red, blue, and combined red and blue lights on pigment contents and SPAD of rice seedlings. (**A**): Chlorophyll a, (**B**): chlorophyll b, (**C**): chlorophyll a + b, (**D**): chlorophyll a/b, (**E**): SPAD, CK: white light, R: red light, R3B1: 75% red light + 25% blue light, R1B1: 50% red light + 50% blue light, R1B3: 25% red light + 75% blue light, B: blue light. According to the LSD test, different lowercase letters indicate statistical differences at the 5% level between different light-quality treatments for the same variety.

There were significant differences in the leaf chlorophyll content and SPAD among different rice varieties and light-quality treatments (Figure 4). Among all light-quality treatments, the chlorophyll content and SPAD resulting from combined red and blue light treatment were higher than those of white light, red light and blue light treatments. In addition, the chlorophyll content and SPAD of XZX24 (HZY261) were the highest under R1B3 treatment, and the chlorophyll a, chlorophyll b, chlorophyll a + b, and SPAD values of XZX24 (HZY261) rice seedlings under R3B1 treatment significantly increased by 24.27%, 17.35%, 22.75%, and 14.88% (20.00%, 17.94%, 19.36%, and 22.74%) compared with white-light treatment, respectively. XZX24 (HZY261) showed higher leaf chlorophyll content and SPAD under blue-light rather than red-light treatment.

### 2.7. Chlorophyll Fluorescence

**Figure 5:**
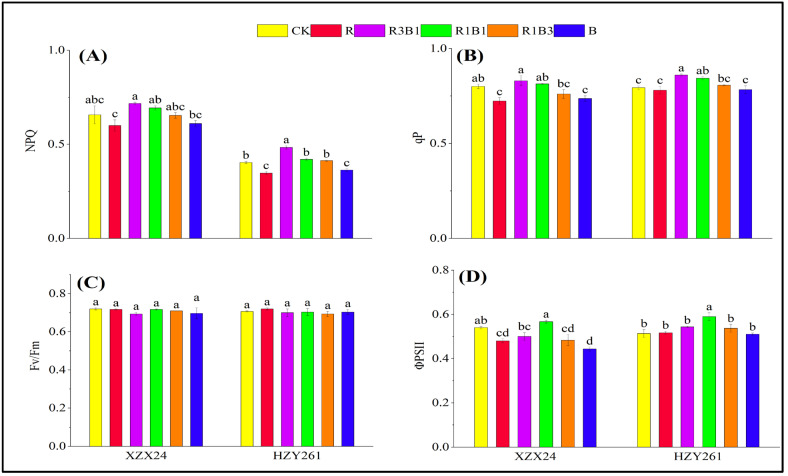
Effects of red, blue, and combined red and blue lights on the chlorophyll fluorescence of rice seedlings. (**A**): NPQ, (**B**): qP, (**C**): Fv/Fm, (**D**): ΦPSII, CK: white light, R: red light, R3B1: 75% red light + 25% blue light, R1B1: 50% red light + 50% blue light, R1B3: 25% red light + 75% blue light, B: blue light. According to the LSD test, different lowercase letters indicate statistical differences at the 5% level between different light-quality treatments for the same variety.

There were significant differences in NPQ, qP, Fv/Fm, and ΦPSII among different rice varieties and light-quality treatments (Figure 5). Among all light-quality treatments, the NPQ and qP under combined red-and-blue-light (R3B1) treatment were higher than those under white light, red light and blue light treatments, and the NPQ and qP of XZX24 (HZY261) rice seedlings under R3B1 treatment significantly increased by 9.14%, 3.75% (19.84%, 8.41%) compared with white-light treatment, respectively. XZX24 (HZY261) showed higher NPQ and qP values in B rather than R; however, the difference was not significant. However, there was no significant difference in the Fv/Fm of XZX24 (HZY261) between treatments (Figure 5C). Among all light-quality treatments, the ΦPSII value under combined red-and-blue-light (R1B1) treatment was higher than those under white light, red light and blue light treatments, and the ΦPSII value of XZX24 (HZY261) rice seedlings under R1B1 treatment significantly increased by 17.26% (14.19%) compared with the white-light treatment.

### 2.8. Photosynthesis

**Figure 6:**
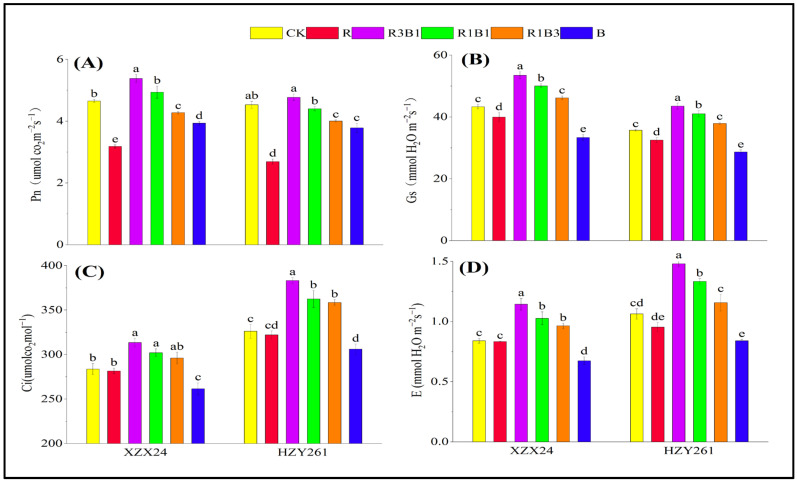
Effects of red, blue, and combined red and blue lights on chlorophyll fluorescence of rice seedlings. (**A**): Pn, photosynthetic rate, (**B**): Gs, stomatal conductance, (**C**): Ci, intercellular CO_2_ concentration, (**D**): E, transpiration rates of leaves, CK: white light, R: red light, R3B1: 75% red light + 25% blue light, R1B1: 50% red light + 50% blue light, R1B3: 25% red light + 75% blue light, B: blue light. According to the LSD test, different lowercase letters indicate statistical differences at the 5% level between different light-quality treatments for the same variety.

There were significant differences in Pn, Gs, Ci, and E among different rice varieties and light-quality treatments (Figure 6), and the Pn and Gs of XZX24 were better than HZY261; nevertheless, the Ci and E of HZY261 were better than XZX24. Red light increased the photosynthetic parameters (Pn, Gs, Ci, and E) of the rice seedlings. Compared with white light treatment, Pn, Gs, Ci and E values of XZX24 (HZY261) seedlings under R3B1 treatment were significantly increased by 15.62%, 23.52%, 10.51% and 36.11% (5.30%, 21.89%, 17.48% and 39.19%), respectively. In addition, XZX24 and HZY261 also showed a higher Pn value under blue-light rather than red-light treatment. However, XZX24 and HZY261 also showed higher Gs, Ci, and E values under red-light rather than blue-light treatment.

### 2.9. Rubisco Activity, Gene Expression

**Figure 7:**
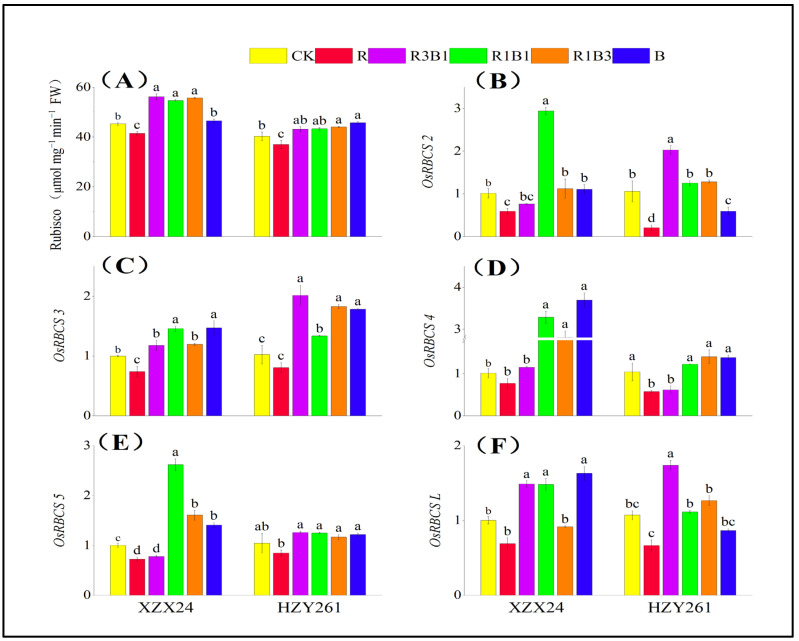
Effects of red, blue, and combined red and blue lights on the Rubisco activity and gene expression of rice seedlings. (**A**): Rubisco activity, (**B**): relative expression level of *OsRBCS2*, (**C**): relative expression level of *OsRBCS3*, (**D**): relative expression level of *OsRBCS4*, (**E**): relative expression level of *OsRBCS5*, (**F**): relative expression level of *OsRBCSL*, CK: white light, R: red light, R3B1: 75% red light + 25% blue light, R1B1: 50% red light + 50% blue light, R1B3: 25% red light + 75% blue light, B: blue light. According to the LSD test, different lowercase letters indicate statistical differences at the 5% level between different light-quality treatments for the same variety.

There were significant differences in the Rubisco activity among different rice varieties and light-quality treatments (Figure 7A), and the Rubisco activity of XZX24 was better than HZY261. Combined-light treatment promoted the Rubisco activity of rice seedlings. Compared with the white-light treatment, the Rubisco activity of XZX24 (HZY261) rice seedlings under R3B1, R1B1, and R1B3 treatments significantly increased by 24.03%, 20.63%, and 23.10% (7.12%, 7.45%, and 9.30%), respectively. In addition, XZX24 (HZY261) also showed higher Rubisco activity under blue-light rather than red-light treatment.

As shown in Figure 7B–F, different light qualities significantly affect the expression levels of *OsRBCS2*, *OsRBCS3*, *OsRBCS4*, *OsRBCS5*, and *OsRBCSL*. Herein, a varying-degrees downregulation of *OsRBCS2*, *OsRBCS3*, *OsRBCS4*, *OsRBCS5*, and *OsRBCSL* expressions of XZX24 and HZY261 was observed under single-red-light treatment relative to CK (Figure 7B–F). At the same time, a varying-degrees upregulation of *OsRBCS2*, *OsRBCS3*, *OsRBCS4*, *OsRBCS5*, and *OsRBCSL* expressions of XZX24 was observed under single-blue-light treatment relative to the white-light treatment; a varying-degrees upregulation of *OsRBCS3*, *OsRBCS4*, and *OsRBCS5* expressions of HZY261 was observed under the single-blue-light treatment relative to the white-light treatment. Relative to CK, *OsRBCS2*, *OsRBCS3*, *OsRBCS4*, *OsRBCS5*, and *OsRBCSL* expressions of XZX24 (HZY261) were downregulated and increased by 41.66%, 21.63%, 23.25%, 27.74%, and 31.20% (80.29%, 21.28%, 44.63%, 19.00%, and 37.72%) under single-red-light treatment, respectively. Relative to the single-red-light treatment, *OsRBCS2*, *OsRBCS3*, *OsRBCS4*, *OsRBCS5*, and *OsRBCSL* expressions of XZX24 (HZY261) were upregulated and increased by 87.16%, 99.03%, 376.33%, 94.20%, and 136.05% (184.79%, 121.45%, 139.55%, 43.95%, and 29.94%) under single-blue-light treatment, respectively. In addition, a varying-degrees upregulation of *OsRBCS2*, *OsRBCS3*, *OsRBCS4*, *OsRBCS5*, and *OsRBCSL* expressions of XZX24 and HZY261 was observed under the combined-light treatment relative to single-red-light treatment (Figure 7B–F).

### 2.10. Heat Map Analysis

**Figure 8:**
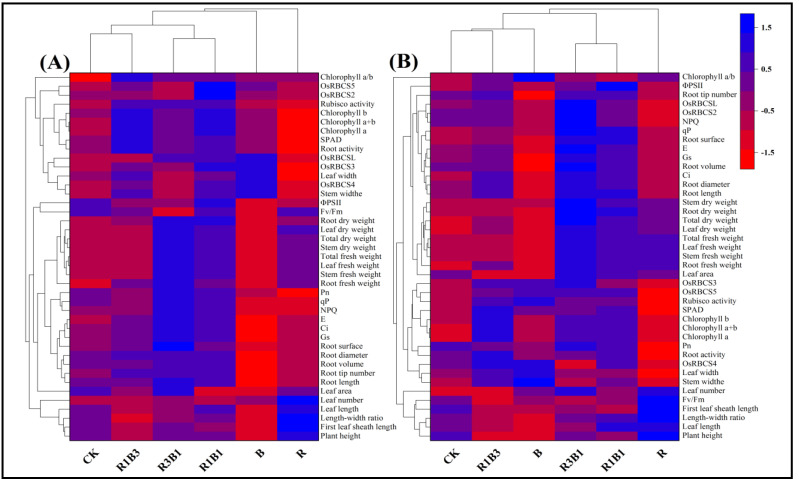
Responses of rice seedlings to white, red, blue, and red and blue combined lights by cluster heat map analysis. (**A**): XZX24, (**B**): HZY261, CK: white light, R: red light, R3B1: 75% red light + 25% blue light, R1B1: 50% red light + 50% blue light, R1B3: 25% red light + 75% blue light, B: blue light. The results are visualized using a false color scale, with blue indicating an increase in response parameters and red indicating a decrease in response parameters.

A heat map of the integrated measurement parameter response provided a comprehensive view of the effects of light quality on the photomorphogenesis and photosynthetic characteristics of the rice seedlings (Figure 8). Under combined-light treatment, most of the measured parameters of XZX24 (HZY261) were significantly larger than that of single-light treatment. In addition, red and blue light treatments showed opposite responses for most measurement parameters.

Among the XZX24 varieties, CK and R1B3 clusters were the closest in terms of measured parameter response, and R3B1 and R1B1 clusters were closest in terms of measured parameter response (Figure 8A). In addition, the CK, R1B3, R3B1, and R1B1 clusters were equally distant from the B cluster. At the same time, cluster B was considerably separated from the other four clusters (CK, R1B3, R3B1, and R1B1): blue light reduced ΦPSII, Fv/Fm, fresh weight (root, stem, leaf, total), dry weight (root, stem, leaf, total), Ci, Gs, root length, and root volume, and increased leaf and stem widths compared to CK, R1B3, R3B1, and R1B1, contributing to separating the B cluster from the others. In addition, cluster R was the furthest away from clusters CK, R1B3, R3B1, R1B1, and B, and cluster R was further away from the other five clusters: red light reduced Pn, gene expression (*OsRBCS2*, *OsRBCS3*, *OsRBCS4*, *OsRBCS5*, and *OsRBCSL*), chlorophyll b, SPAD, and root activity, and increased plant height, first leaf sheath length, leaf length, length–width ratio, and leaf number compared to the other five treatments, helping to distinguish the R clusters from the other clusters.

In the HZY261 variety, the measured parameter responses of the CK and R1B3 clusters were the closest, with equal distances to cluster B (Figure 8B). At the same time, cluster B was considerably separated from the other two clusters (CK and R1B3): blue light reduced ΦPSII, root tip number, gene expression (*OsRBCS2* and *OsRBCSL*), NPQ, qP, root surface, Gs, E, plant height, root volume, Ci, root diameter, root length, fresh weight, first leaf sheath length, leaf length, and dry weight, and increased gene expression (*OsRBCS3*, *OsRBCS4*, and *OsRBCS5*), root activity, SPAD, and leaf width compared to CK and R1B3, contributing to separating the B cluster from the others (CK and R1B3). Furthermore, the R3B1 and R1B1 clusters were the closest to each other in terms of measured parameter responses, and were equidistant from cluster R. Red light reduced gene expression (*OsRBCS2*, *OsRBCS3*, *OsRBCS4*, *OsRBCS5*, and *OsRBCSL*), NPQ, Rubisco activity, SPAD, Pn, chlorophyll a, chlorophyll b, root activity, leaf width, and stem width, and increased plant height, leaf length, length–width ratio, first leaf sheath length, and Fv/Fm compared to R3B1 and R1B1, contributing to separating the R cluster from the others (R3B1 and R1B1).

## 3. Discussion

### 3.1. Red and Blue Lights Can Regulate the Early Photomorphogenesis of Rice

Light quality plays a very important role in regulating the differentiation and growth of crop cells and the photosynthesis of leaves. Therefore, external morphology is one of the intuitive manifestations of crop adaptability to light environment. Two rice varieties XZX24 (HZY261) were used as materials to study the effects of different light-quality treatments on seedling growth. The results of the present study are consistent with the results of previous research [11] that determined that plant height, leaf length, leaf area, leaf number, root length, and root volume of XZX24 (HZY261) under red-light treatment were greater than those under blue-light treatment (Figure 1A,B; Table 1 and Table 2). However, the leaf width, stem width, and root activity values of XZX24 and HZY261 under blue-light treatment were greater than those under red-light treatment (Figure 1C and Figure 2; Table 1) [27,28]. Meanwhile, the fresh and dry weights of XZX24 (HZY261) under red-light treatment were also higher than those under blue-light treatment (Figure 3) [29,30]. This shows that red light can promote rice seedling elongation and dry matter accumulation, which is consistent with the results for cotton [31] and chrysanthemum [32]. This result is contrary to the findings of Gao [30,33], which show that blue light can promote plant growth and dry matter accumulation. The differences in the study results may be related to the different spectral absorption ranges of different plant materials and varieties, and may also be related to other factors, such as temperature, air relative humidity, irrigation, fertilizer, and crop management. The effects of comprehensive environmental factors on the morphogenetic characteristics of rice seedlings need to be studied further.

In comparison to monochrome red and blue lights, the combination of red and blue lights is more conducive to plant morphogenesis [11,25,34]. R3B1 treatment significantly increased the leaf width, root length, root volume, and fresh and dry weights of XZX24 (HZY261) (Table 1 and Table 2; Figure 2 and Figure 3). The experimental results show that the correct amount of blue light helps to promote the main effect of red light; however, too much blue light can counteract the effect of red light. Previous studies have reported that red light promotes tomato stem elongation, and the addition of a certain amount of blue light can reverse this phenomenon [24,35]. These results indicate that the regulatory role of light in plant morphogenesis is complex [36,37]. In addition, the leaf parameters, growth parameters, and fresh and dry weights of XZX24 were greater than those of HZY261, indicating that XZX24 had better adaptability and higher response mechanisms to light quality. In addition, the strength of rice seedlings directly affected the growth of rice after transplantation; however, the final yield of rice not only depended on the quality of rice seedlings, but also had a close relationship with the ecological environment for rice growth. Whether the influence of light quality on the quality of rice seedlings leads to a difference in the yield needs to be studied further.

### 3.2. Effects of Red and Blue Lights on Photosynthetic Pigments and Chlorophyll Fluorescence

Photosynthetic pigment is the material basis of the photosynthesis of crops, which directly determines the ability of plants to absorb and fix light energy [38]. In addition, due to the different absorption capacities of red and blue pigments, the formation of photosynthetic pigments in plants is different [39,40]. The results of this study show that the chlorophyll content of XZX24 (HZY261) is higher in single-blue-light rather than in single-red-light conditions, and is higher in combined red-and-blue-light conditions than under single-light treatment, and relatively higher under R1B3 treatment (Figure 4A–C,E). This research result is consistent with the results of the study of light quality on n solanum melongena [11] and solanum tuberosum [41]. These observations may reflect the feedback regulation mechanism mediated by blue-light receptors during plant photosynthesis, that is, the light-induced transformation of photosynthetic pigment synthesis and accumulation in plants through the photosensitive pigment system, and the positive effect of the red–blue-light combination on plant morphogenesis.

Photosynthetic fluorescence parameters are an effective means to study the photosynthetic performance of crops at present and have been applied to monitor the photosynthetic performances of various crops [42,43]. The NPQ of XZX24 values were better than HZY261 under each light-quality treatment, which may be why the reason why the values of the leaf parameters, growth parameters, and fresh and dry weighs of XZX24 were higher than HZY261. The ΦPSII value of XZX24 (HZY261) under combined-light treatment was higher than that during single-light treatment, and they achieved the higher ΦPSII values under R3B1 treatment (Figure 5D). This indicated that the absorption efficiency of light energy was similar and R3B1 treatment was the best [44]. In addition, the NPQ value during R3B1 treatment was also relatively higher than for the other treatments; this indicated that the rice seedlings could better consume excess light energy in the form of heat energy during R3B1 treatment to maintain a faster growth rate [45]. However, the improvement of chlorophyll fluorescence by the combination of red and blue lights was caused by the complex physiological mechanism of crop photosynthesis, rather than a simple additive effect. The related law of the combined effect of the combination of red and blue lights on photosynthesis still needs further study.

### 3.3. Effects of Red and Blue Lights on Photosynthesis, Rubisco Activity, and Gene Expression

Plant growth depends on photosynthesis to produce organic matter, and the intensity of photosynthesis is determined by the amount of solar energy fixed by chloroplasts in plant leaves, which is closely related to light quality [46,47,48]. Different light-quality amounts can regulate physiological processes, such as gas exchange and chlorophyll formation, in plant leaves [49], and the photoreceptors (photochromin and cryptochrome) and chloroplasts of leaf cells can regulate stomatal volume and stomatal number according to different light textures [50,51,52]. This study showed that XZX24 and HZY261 had the highest values for photosynthetic parameters under R3B1 treatment (Figure 5). The photosynthetic parameter of XZX24 (HZY261) under combined-light treatment was significantly higher than that under single-light-treatment conditions. This was similar to the results for cucumbers [20] and eggplants [11]. In addition, XZX24 and HZY261 showed higher Pn values under blue- rather than red-light treatment. However, XZX24 and HZY261 also showed higher Gs, Ci, and E values under red- rather than blue-light treatment. In conclusion, the lack of red or blue light caused a decrease in the photosynthetic efficiency of the rice seedlings, and the appropriate high proportion of red light could promote the photosynthesis of the rice seedlings. In other words, the combined red and blue lights were more effective than monochromatic light to improve and maintain the higher photosynthetic performance of the plants [46,48]. In the spectrum of total solar radiation, only the radiation in the visible range could produce effective light waves for the photosynthesis of the plants. At the same time, the photosynthetic rate of plant leaves was different when plants were cultured with monochromatic-light sources in different bands. According to the spectral analysis of plant photosynthesis, peak plant photosynthesis was attained in the blue (440 nm) and red (620 nm) regions, and the height of the red wave peak was about 1.4 times that of the blue wave peak. Red and blue lights are the main light sources of plant photosynthesis [53,54]. In addition, it was found in the study that the transpiration and stomatal conductivity of plants growing under combined-light treatment were significantly increased compared with CK, which may have been due to the synergistic effect of the combined application of red and blue lights, so as to improve the photosynthetic performance of the rice seedlings.

The Rubisco activity was directly determined by the expression of photosynthetic related genes (*OsRBCS2*, *OsRBCS3*, *OsRBCS4*, *OsRBCS5*, and *OsRBCSL*). The enzyme activity of Rubisco directly affected the Pn of the rice seedlings. With the enhancement of Pn, the accumulation of carbon metabolites increased, and the plants could accumulate more dry matter. When combining Rubisco activity and gene expression, the results of this study show that XZX24 (HZY261) presented the greatest Rubisco activity in B rather than R during all light-quality treatments (Figure 7A), and *OsRBCS2*, *OsRBCS3*, *OsRBCS4*, *OsRBCS5*, and *OsRBCSL* expressions under B-treatment conditions were higher than R treatment (Figure 7B–F), which may have been due to red light inhibiting the expression of these genes but activating the expression of other genes related to Rubisco activity. The Rubisco activity of XZX24 was not significantly different under combined-light-treatment conditions and was significantly higher than during single-light treatment [55,56]. Nevertheless, the Rubisco activity of HZY261 was not significantly different under combined-light-treatment conditions and was significantly higher than during white-and red-light treatments. HZY261 also presented greater Rubisco activity during single-blue-light treatment rather than combined-light treatment, and the difference was not significant. In addition, XZX24 and HZY261 also showed higher *OsRBCS2*, *OsRBCS3*, *OsRBCS4*, *OsRBCS5*, and *OsRBCSL* expression levels during combined-light treatment rather than single-red-light (R) treatment. It can be observed from the *OsRBCS2*, *OsRBCS3*, *OsRBCS4*, *OsRBCS5*, and *OsRBCSL* expression levels that blue light was conducive to the expression of Rubisco-related genes and improved Rubisco activity, so as to improve the photosynthetic intensity of plant leaves. Due to the low number of genes involved in this study and the low match with Rubisco activity, which limited the comparability of the results, the target genes will be screened by genomics and transcriptomics methods in the subsequent research. At the same time, the following step will be to use gene editing and other techniques to construct light-sensitive rice materials to provide a more theoretical basis for rice seedling development.

## 4. Materials and Methods

### 4.1. Plant Materials and a Description of the Experiment

The experiment was conducted in an artificial climate chamber at Hunan Agricultural University from 10 April to 18 October 2022. The germinated rice (XZX24 and HZY261) seeds (n = 3 grains/hole) were seeded on a seedling tray, and 80% of the seedlings were exposed to different light treatments. The six light treatments’ growth conditions were as follows: (1) CK, white light; (2) R, red light; (3) R3B1, 75% red light+ 25% blue light; (4) R1B1, 50% red light + 50% blue light; (5) R1B3, 25% red light+ 75% blue light; (6) B, blue light. Daytime: temperature = 25 °C, light intensity = 100 μmolm^−2^s^−1^, and photoperiod = 12 h. Night: temperature = 15 °C. Central wavelength of red light, 665 nm, and central wavelength of blue light, 450 nm.

### 4.2. Morphological Indicators and Root Activity Analysis

The plant height, first leaf sheath length, and stem base width of the rice seedlings were measured after 28 days of light culture. At the same time, an LA-S analysis system (LA-S, Hangzhou Wanshen Testing Technology Co., Ltd., Hangzhou, China) was used to analyze the roots and leaves of the rice seedlings and to obtain the relevant data for the roots and leaves. In addition, 50 plants were randomly selected from each replicate to measure the fresh and dry weights of each part of the rice seedlings.

The root activity of the rice seedlings was tested by TTC reduction method [57], by obtaining 0.5 g of fresh roots of rice seedlings, adding 5 mL of TTC (0.4%) and 5 mL of phosphoric acid buffer (PH = 7.0), keeping them in a dark place at 37 °C for 1 h, then adding 2 mL of sulfuric acid (1 mol L^−1^) to stop the reaction, keeping them for 15 min, pouring out sulfuric acid to preserve the roots, adding 20 mL of methanol, and keeping them at 35 °C for 6 h. The root activity was measured at 485 nm with a spectrophotometer (Hitachi, Tokyo, Japan, U-2900) and the reduction amount of tetrazolium was determined by checking the standard curve.

### 4.3. Chlorophyll Content and SPAD of Leaves

After cleaning the fresh rice seedling leaves with sterile distilled water, 0.1 g of chopped leaves was weighed and placed in a 1:1 (*v*/*v*) mixture of 10 mL 80% acetone and anhydrous ethanol and treated in a dark environment for 12 h [21]. The absorbance of the extract was determined by ultraviolet spectrophotometer (Shimadzu, Tokyo, Japan) at 645 nm (OD645) and 663 nm (OD663) with 80% acetone as a blank. The chlorophyll content was calculated as follows: chlorophyll a content (mg g^−1^) = 12.7 × OD663 − 2.69 × OD645; chlorophyll b content (mg g^−1^) = 22.9 × OD645 − 4.86 × OD663. In addition, the SPAD of the second leaf from the apex was assessed by Chlorophyll Meter (Konica minolta, Tokyo, Japan).

### 4.4. Chlorophyll Fluorescence

Chlorophyll fluorescence was measured by a FluorPen 110/D (PSI, Photon Systems Instruments, Drásov, Czech Republic). The Oxborough [58] method was used to detect chlorophyll fluorescence after 21 days of cultivation of the rice seedlings under different light-quality conditions. For the calculation of the chlorophyll fluorescence parameters (NPQ, qP, Fv/Fm, and ΦPSII), please refer to Ren [59].

### 4.5. Photosynthetic Parameter Analysis

After the rice seedlings were cultured in different light conditions for 21 days, the Pn, Gs, Ci, and E values of the rice seedlings were measured by an infrared gas analyzer (LI-6400, Li-COR, Lincoln, OR, USA) [60].

### 4.6. Rubisco Activity and Gene Expression

After 21 days of cultivation of the rice seedlings under different light conditions, the activity of Rubisco was determined by a second fully unfolded leaf. The activity of Rubisco was determined by the production of 3-phosphoglyceric acid and the oxidative coupling of NADH at 25 °C using a spectrophotometric method described by Sun [61].

We examined the expression levels of Rubisco-related genes (*OsRBCS2*, *OsRBCS3*, *OsRBCS4*, *OsRBCS5*, and *OsRBCSL*) in leaves under different light-quality conditions. The specific primers of Rubisco-related genes and the internal reference gene *OsActin3* are detailed in Table 3. For the determination and calculation of the relative expression, please refer to Ren description [62].

## 5. Conclusions

The combined application of red and blue lights promoted the growth of rice seedlings to varying degrees; enhance photosynthesis by increasing the seedling leaf area, chlorophyll content, and chlorophyll fluorescence; improve root characteristics by increasing the root number, root volume, and root activity; and thus increase the dry matter accumulation of the rice seedlings. In addition, the combination of red and blue lights regulated the expression of genes related to photosynthesis in rice leaves, affected the activity of the Rubisco enzyme, and then affected the photosynthesis of the rice seedlings. These results indicate that red and blue lights have direct synergistic effects, which can regulate the growth of rice seedlings and promote the morphogenesis of rice seedlings. The combined application of red and blue lights can be used to supplement the light in rice-factory seedling raising.

## Figures and Tables

**Table 1:** Effects of red, blue, and combined red and blue lights on leaf growth of rice seedlings.

Variety	Treatments	Leaf Length (cm)	Leaf Width (mm)	Length-Width Ratio	Leaf Area (cm^3^)	Leaf Number (No. Plant^−1^)
XZX24	CK	7.26 ± 0.11 c	3.52 ± 0.03 c	20.64 ± 0.51 b	1.4 ± 0.03 b	2.86 ± 0.04 b
	R	8.57 ± 0.06 a	3.11 ± 0.04 d	27.58 ± 0.40 a	1.27 ± 0.02 c	3.34 ± 0.07 a
	R3B1	6.45 ± 0.04 d	3.42 ± 0.03 c	18.86 ± 0.30 c	1.52 ± 0.03 a	2.90 ± 0.09 b
	R1B1	7.68 ± 0.06 b	3.85 ± 0.07 b	19.98 ± 0.25 b	1.02 ± 0.02 e	2.98 ± 0.10 b
	R1B3	5.78 ± 0.04 e	4.05 ± 0.04 a	14.27 ± 0.19 d	1.16 ± 0.02 d	2.90 ± 0.00 b
	B	5.44 ± 0.06 f	4.09 ± 0.04 a	13.3 ± 0.20 d	1.01 ± 0.02 e	3.02 ± 0.08 b
HZY261	CK	7.01 ± 0.05 b	3.75 ± 0.04 b	18.71 ± 0.11 c	1.26 ± 0.02 ab	2.74 ± 0.04 c
	R	8.33 ± 0.04 a	3.26 ± 0.04 c	25.53 ± 0.24 a	1.41 ± 0.02 a	3.06 ± 0.04 a
	R3B1	6.79 ± 0.14 b	3.65 ± 0.04 b	18.62 ± 0.52 c	1.23 ± 0.12 b	3.02 ± 0.05 ab
	R1B1	8.02 ± 0.09 a	3.77 ± 0.10 b	21.29 ± 0.49 b	1.32 ± 0.02 ab	2.90 ± 0.00 b
	R1B3	5.97 ± 0.48 c	4.15 ± 0.16 a	14.43 ± 1.20 d	1.01 ± 0.01 c	2.74 ± 0.04 c
	B	5.53 ± 0.22 c	4.34 ± 0.03 a	12.75 ± 0.56 d	0.95 ± 0.02 c	3.02 ± 0.08 ab

Note: According to the LSD test, different lowercase letters indicate statistical differences at the 5% level between different light-quality treatments for the same variety. CK: white light, R: red light, R3B1: 75% red light + 25% blue light, R1B1: 50% red light + 50% blue light, R1B3: 25% red light + 75% blue light, B: blue light.

**Table 2:** Effects of red, blue, and combined red and blue lights on root growth of rice seedlings.

Variety	Treatments	Root Length (cm)	Root Surface Area (cm^2^)	Root Volume (cm^3^)	Root Diameter (mm)	Root Tip Number (No. Plant^−1^)
XZX24	CK	62.89 ± 0.28 c	8.58 ± 0.09 d	0.28 ± 0.02 b	1.18 ± 0.01 a	82.17 ± 0.93 b
	R	54.80 ± 1.05 d	7.39 ± 0.07 e	0.24 ± 0.01 c	0.96 ± 0.02 b	69.17 ± 0.60 c
	R3B1	71.55 ± 1.69 a	11.29 ± 0.17 a	0.31 ± 0.01 a	1.24 ± 0.01 a	96.00 ± 1.04 a
	R1B1	68.29 ± 0.22 b	9.60 ± 0.20 b	0.29 ± 0.01 b	1.22 ± 0.01 a	93.33 ± 1.42 a
	R1B3	65.79 ± 0.48 b	9.02 ± 0.10 c	0.29 ± 0.00 b	1.21 ± 0.04 a	93.83 ± 0.60 a
	B	49.90 ± 1.00 e	6.86 ± 0.09 f	0.19 ± 0.02 d	0.83 ± 0.01 c	60.33 ± 1.20 d
HZY261	CK	42.86 ± 0.26 c	6.54 ± 0.06 c	0.21 ± 0.02 c	0.98 ± 0.04 c	83.17 ± 2.95 b
	R	40.44 ± 0.30 d	6.41 ± 0.20 c	0.19 ± 0.01 d	0.85 ± 0.01 d	66.17 ± 0.67 c
	R3B1	50.15 ± 0.34 a	13.00 ± 0.31 a	0.23 ± 0.02 b	1.22 ± 0.02 b	91.00 ± 0.58 a
	R1B1	51.19 ± 0.88 a	13.46 ± 0.30 a	0.26 ± 0.01 a	1.32 ± 0.02 a	92.17 ± 2.03 a
	R1B3	47.38 ± 0.82 b	8.55 ± 0.16 b	0.22 ± 0.02 bc	1.19 ± 0.01 b	93.17 ± 1.30 a
	B	37.85 ± 0.12 e	5.50 ± 0.09 d	0.16 ± 0.00 e	0.78 ± 0.01 d	50.83 ± 1.42 d

Note: According to the LSD test, different lowercase letters indicate statistical differences at the 5% level between different light-quality treatments for the same variety. CK: white light, R: red light, R3B1: 75% red light + 25% blue light, R1B1: 50% red light + 50% blue light, R1B3: 25% red light + 75% blue light, B: blue light.

**Table 3:** Primer sequences of Rubisco-related genes and *OsActin3*.

Gene	Accession No.	Up-Primer (5′-3′)	Down-Primer (5′-3′)
*OsActin3*	Os03g0718100	CCACTATGTTCCCTGGCATT	GTACTCAGCCTTGGCAATCC
*OsRBCS2*	AK121444	CAGCAAGGTCGGATTCGTCT	CACACGAAACAAGGTGGGAG
*OsRBCS3*	AK068555	TTCCAAGGGCACAAGTCCAC	TAGGCAGCAATCCACCAGC
*OsRBCS4*	AK068266	GTGGATGAGTAAAGGGACAA	CAGGAGTAAATCAAGAGCGT
*OsRBCS5*	AK099574	TACCTGCTCCGGTCCAAATG	TCAAAGCTGCCAAAACCGAA
*OsRBCSL*	OrsajCp033	CCCCGGAGTACGAAACCAAG	GAGGCGGACCTTGGAAAGTT

## Data Availability

The data used to support the findings of this study are available from the corresponding author upon request.

## Supplementary Materials

The supporting information can be downloaded at: https://www.mdpi.com/article/10.3390/ijms241411333/s1.
