# Supplementary material for: Photomorphogenesis and Photosynthetic Traits Changes in Rice Seedlings Responding to Red and Blue Light
Source: Int J Mol Sci. 2023 Jul 12;24(14):11333. doi: 10.3390/ijms241411333 (PMC10378807; doi:10.3390/ijms241411333)
Supplement: Supplementary file 1 [file ijms-24-11333-s001.zip › ijms-2483348-supplementary.pdf]

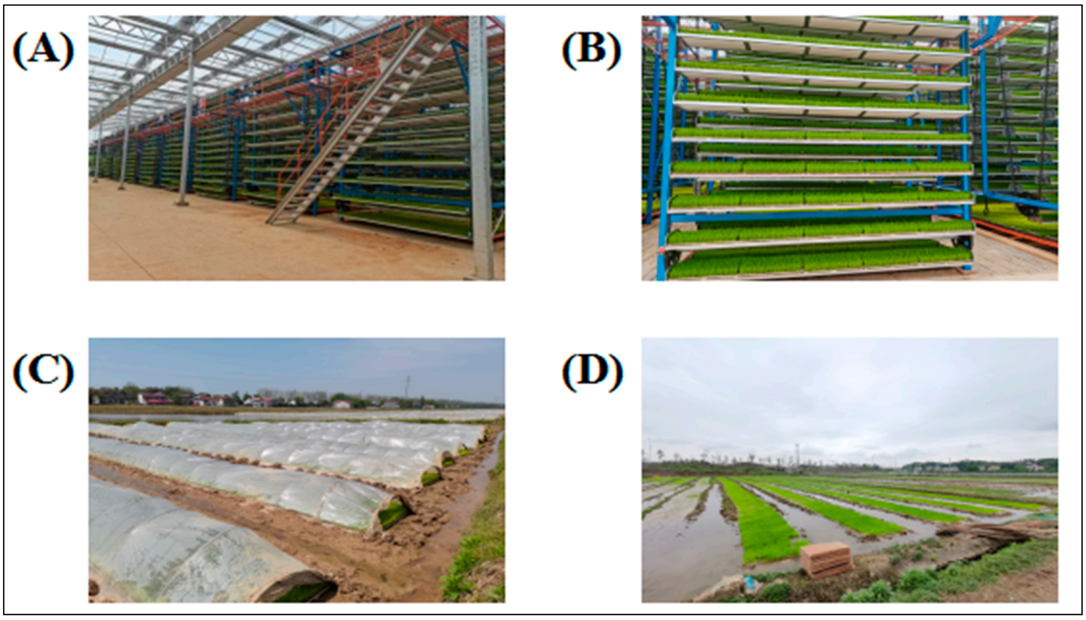

Figure S1 Two types of rice seedling raising modes. (A), (B): Plant factory seedling raising; (C), (D): Traditional rice seedling.
